# Case report: Xanthogranulomutous pyelonephritis presenting as “Wilms’ tumor”

**DOI:** 10.1186/s12894-016-0155-5

**Published:** 2016-07-07

**Authors:** Shakilu Iumanne, Aika Shoo, Larry Akoko, Patricia Scanlan

**Affiliations:** Muhimbili University of health and Allied Sciences, P.O. Box 65001, Dar es Salaam, Tanzania; Muhimbli National Hospital, P.O. Box 65000, Dar es Salaam, Tanzania; College of Health Sciences, University of Dodoma, Box 339, Dodoma, Tanzania

**Keywords:** Xanthogranulomatous pyelonephritis, Wilms’ tumor, Pseudotumor

## Abstract

**Background:**

Xanthogranulomatous pyelonephritis (XGP) is a rare renal tumor that arises as a complication of chronic obstructive pyelonephritis of uncertain etiology. It is primarily an adult tumor seen occasionally in children associated with urinary tract obstruction due to congenital urological anomalies, nephrolithiasis, and recurrent urinary tract infections. Radiologically, it may show neoplastic features such as those seen in common pediatric renal malignancies like wilms’ tumor and renal cell carcinoma. This overlap in radiological manifestation frequently leads to misdiagnosis and delay in appropriate intervention. We report a case of a 3 years old boy who presented with history of recurrent urinary tract infections and a left renal mass initially thought to be Wilms’ tumor.

**Case presentation:**

We present a case of a 3 years old boy admitted to the Pediatric oncology unit at Muhimbli National Hospital in Dar es Salaam, Tanzania with one year history of recurrent fever and urinary tract infection signs and symptoms refractory to antibiotic therapy. He was eventually found to have a left kidney mass detected at the District hospital by abdominal ultrasound performed to evaluate a flank mass that was felt by his mother. He was then referred to our unit for a suspicion of Wilms’ tumor which finally turned out  to be a left kidney Xanthogranulomatous pyelonephritis.

He underwent a successful left nephrectomy and was discharged from hospital in a stable clinical condition and remains asymptomatic at the time of submission of this case report.

**Conclusion:**

This case report underscores the need for clinicians attending a febrile child with a renal mass that can be confused with common pediatric renal malignancies such as Wilms’ tumor to broaden their differential diagnosis. The case also underlines the significance of individualized patient evaluation because this patient would have otherwise received preoperative chemotherapy under the International Society of Pediatric Oncology (SIOP) guidelines if the diagnosis of Wilms tumor was not ruled out.

## Background

Xanthogranulomatous pyelonephritis is a rare renal tumor of uncertain etiology believed to occur as a complication of chronic obstructive pyelonephritis with bacterial superinfection. It is more of an adult disease with peak age at 5th to 6th decade of life, occasionally seen in children associated with urinary tract obstruction mostly from congenital urological malformations such as posterior urethral valve, nephrolithiasis, and recurrent urinary tract infections [1]. Radiologically, it may display similar neoplastic features as those seen with common renal malignancies such as wilms’ tumor and renal cell carcinoma appearing as a flank mass invading neighboring structures [2]. This overlap in radiological characteristics frequently leads to misdiagnosis and delay in appropriate interventions. Surgery and antibiotic therapy are the mainstay of treatment for this rare urological condition with open radical nephrectomy and resection of affected neighbouring structures being the standard of treatment but laparascopic surgery has been used to treat some cases [3]. We report a case of a 3 years old boy presenting with history of recurrent urinary tract infections and a left renal mass that was finally diagnosed as Xanthogranulomatous pyelonephritis.

**Table 1:** Summarized laboratory investigation results

Total white cell count (/μl)	Neutrophil count (/μl)	Lymphocyte count (/μl)	Monocyte count (/μl)	Hemoglobin (g/dl)
12700	6770	4227	700	6.17
Redblood cell (/μl)	MCV (fl)	MCH (pg/cell)	Plateletcount (/μl)	RDW (%)
2680	57.5	16.7	608	16
Serum Chemistry				
Serum creatinine (μmol/L)	Urea (μmol/L)	LDH (U/L)	Sodium (mmol/L)	Calcium (mmol/L)
41.6 μmol/L	5.4	281	133	1.25
Urine analysis				
Leukocytes/ (hpf)	Nitrites	Protein	pH	Specific gravity
20	1^+^	2^++^	6.8	1.005

## Case presentation

A 3 years old boy presented with history of recurrent fever, episodes of painful urination, increased frequency and haematuria for one year. He had been treated with several courses of antibiotics for urinary tract infections with temporary relief of symptoms before recurrence. At initial presentations several urine analysis using dipstics were performed at the local dispensary and reported to show features of urinary tract infections but no further work up such as urine culture, voiding cyctourethrogram or abdominal ultrasound was performed. Three months before admission to our unit, his mother had felt a mass on his left flank and an abdominal ultrasound done at the nearby District Hospital suspected Wilms’ tumor and the patient was referred to our unit for further evaluation. The patient had otherwise attained his developmental mile stones as per age and had no any dysmorphic features.

On examination; the child was febrile with body temperature of 38.8 °C with tenderness on the left lumber region and had a palpable mass on the same side. Laboratory investigations performed are summarised in the concise table below; Table 1.

A repeat abdominal ultrasound showed a left renal mass 7.4X7.1X5 cm with multiple cystic lesions. The renal pelvis was destroyed with some calcific changes but no extension to the neighboring structures were noted. The right kidney was normal in size and echogenicity.

**Fig. 1:**
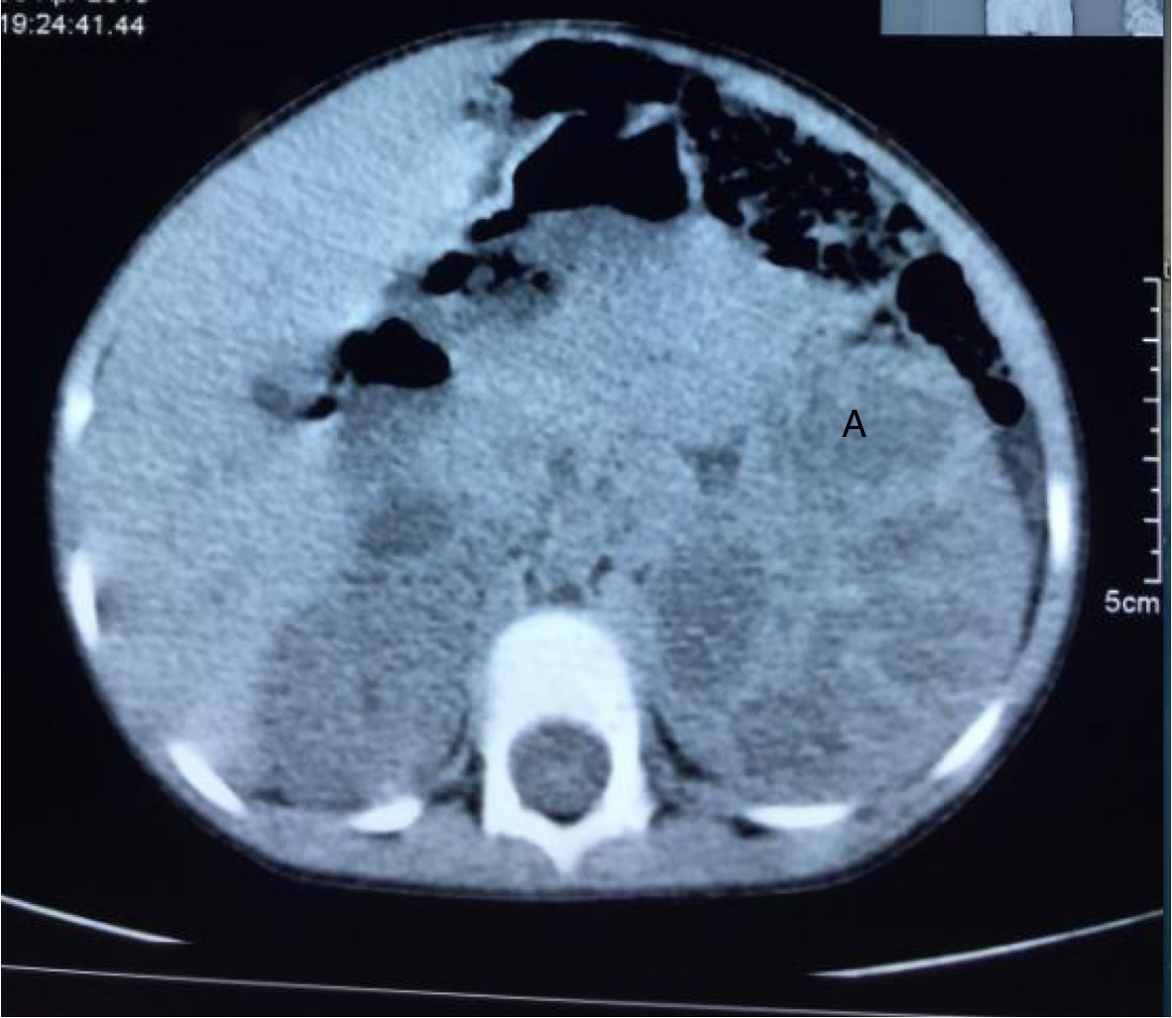
Plain abdominal CT-scan showing an enlarged left kidney with multiple cystic components (A) and calcifications

**Fig. 2:**
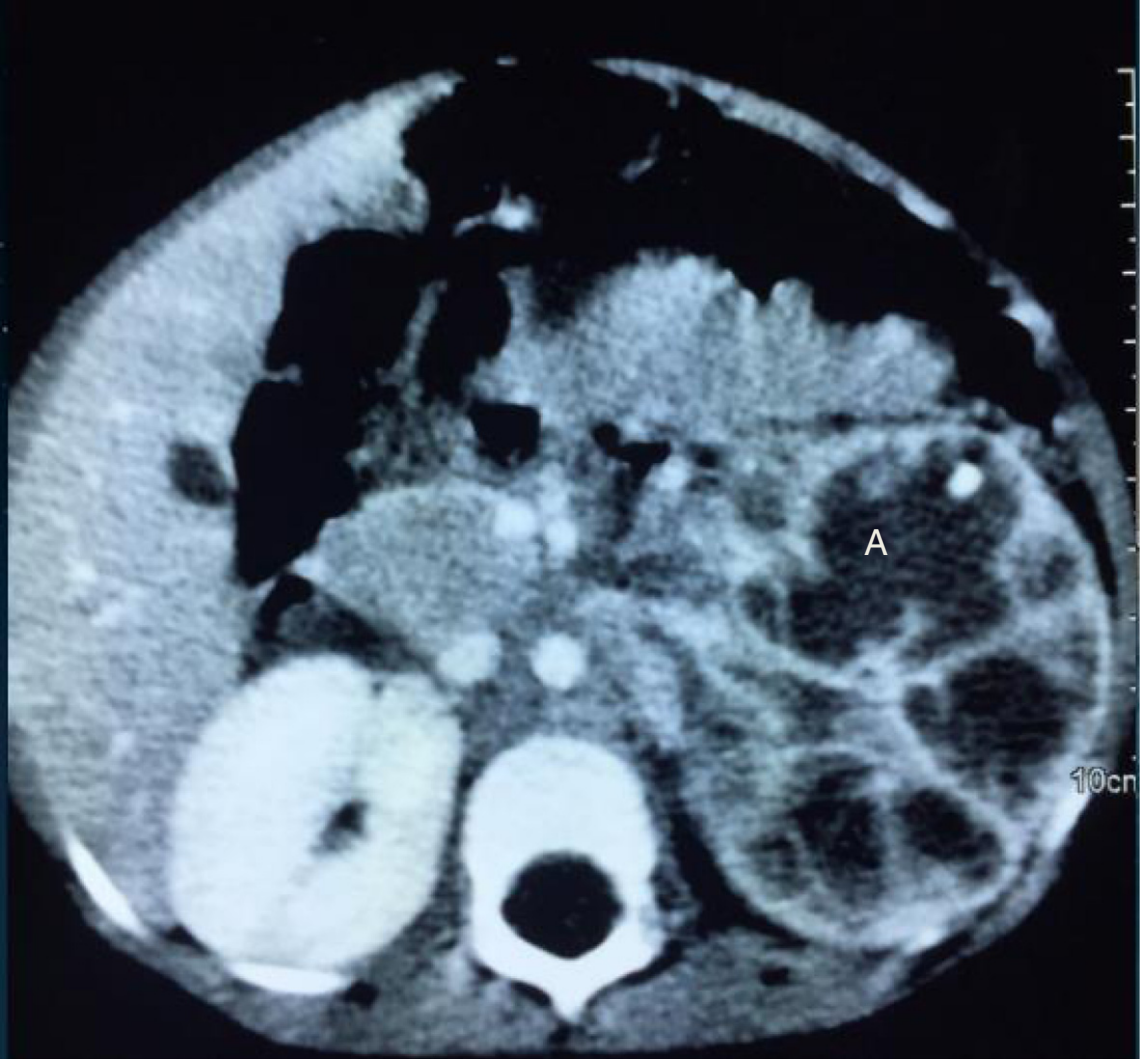
Contrast enhanced abdominal CT-scan showing multiple cysts in the left kidney (A) with peripheral enhancement of residual normal renal tissuesᅟ

Abdominal CT Scan (Figs. 1 and 2) showed an enlarged left kidney measuring 7.9X6.7 cm in size with thinning of the cortices and loss of corticomedullary differentiation, foci of calcifications were also seen and the renal parenchyma was replaced with multiple septations (A). Contrast enhanced CT scans showed areas of peripheral enhancement surrounding non-enhancing cystic foci within the mass. There was no obvious filling defect seen in the renal vein or inferior vena cava. The right kidney was normal in size and shape, urinary bladder displayed normal outline and other abdominal organs  were all normal.

**Fig. 3:**
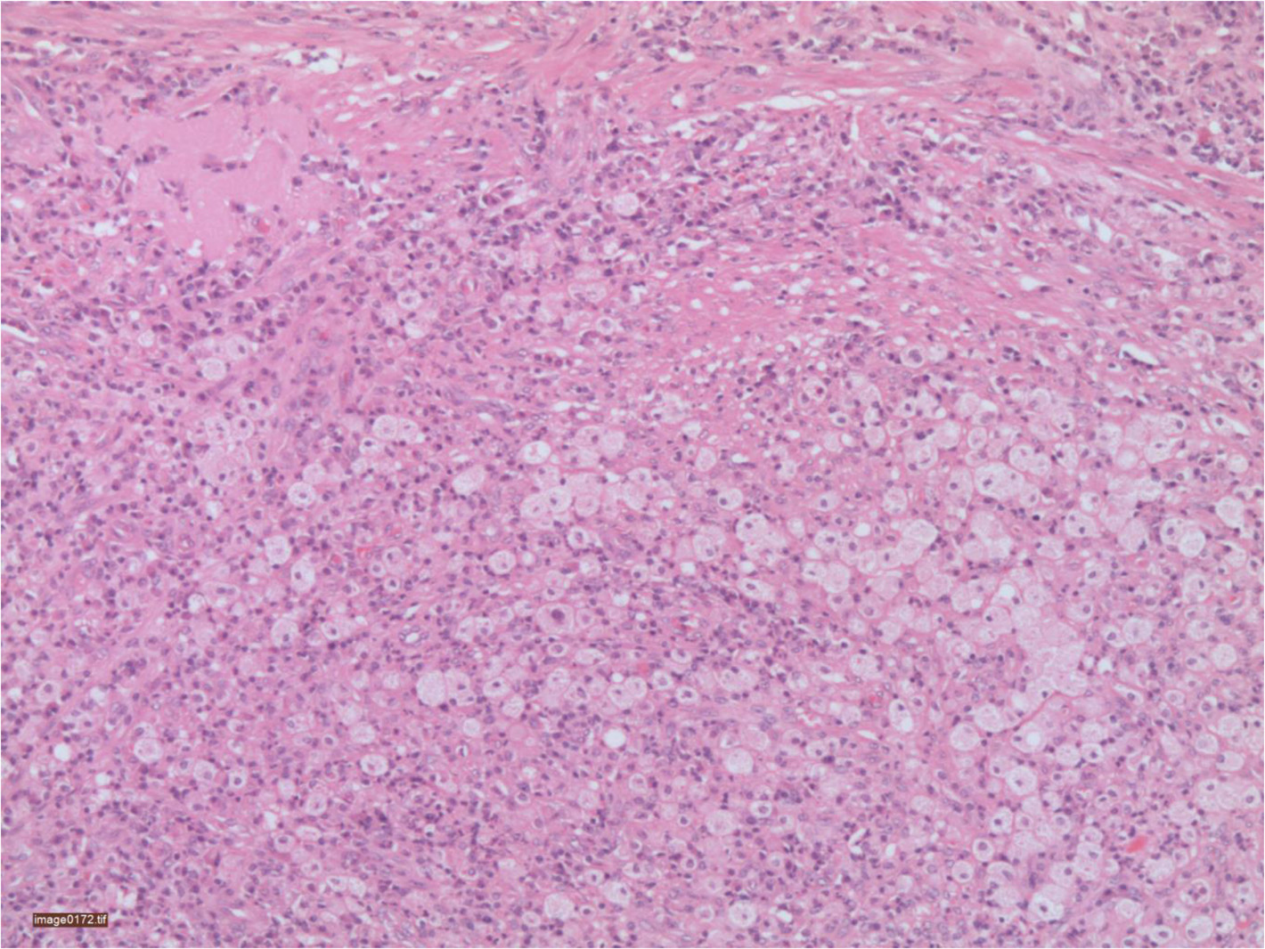
H&E micrograph of a histological section of the mass showing lipid laden macrophages in a mixture of chronic inflammatory cells

Based on the clinical presentation and abdominal CT scan, a clinical diagnosis of Xanthogranulomatous pyelonephritis was made and the patient underwent left radical nephrectomy. Intra-operatively an enlarged yellowish grey left kidney with multiple mesenteric and hilar lymph nodes was seen, radical left nephroureterectomy and hilar lymphnode resection was performed, the right kidney was normal on inspection. The resected tissues were sampled and sent for culture and sensitivity but there was no bacterial growth after 7 days. Histological studies reported macroscopically gross yellowish renal tissues microscopically showing a renal histology infiltrated with foamy lipid laden macrophages in a mixture of chronic inflammatory cells and fibrosis confirming a diagnosis of Xanthogralomatous pyelophritis (Fig. 3).

The patient faired well postoperatively and was kept on a 7 days course of intravenous Tazobactam/piperacillin and discharged home 10 days post nephrectomy. 

## Discussion

Xanthogranulomatous pyelonephritis is a rare chronic obstructive pyelonephritis characterized by infiltration of the renal parenchyma with lipid laden Macrophages and an enlarged non-functional kidney [4]. It is most commonly associated with superinfections by bacteria such as *E.coli, Proteus mirabilis,* and occasionally *Pseudomonas species* [5]. Renal calculi, diabetes mellitus and immunosuppressive conditions are also reported to predispose an individual to this rare renal tumor [6]. The affected kidney is usually non-functional and can be mistaken with common renal neoplasms because it may locally extend to invade adjacent structures such as the psoas muscle, pancreas, spleen and the duodenum.

In most cases, Xanthogranulomatous pyelonephritis involve one kidney but cases of bilateral renal involvement have been published [7, 8]. XGP is predominantly a disease of adults reported to occur in approximately 1 % of pyelonephritis. It is four times more common in females than males with a peak age at fifth and sixth decades of life [9]. XGP in adults affects both kidneys equally but it involves the left kidney more often than the right kidney in children as it was the case for our patient [10]. XGP in children occur more in boys usually from 8 years of age and it is encountered in approximately 16 % of pediatric nephrectomy specimens [11]. Histologically the disease is characterized by renal parenchyma infiltration by lipid laden macrophages and chronic inflammatory cells in line with the histological finding for our patient [12]. The exact mechanism of xanthogranulomatous pyelonephritis (XGP) is unclear, but it is generally agreed that the disease process requires a long-term renal obstruction superimposed with infection by *Proteus* species, *Escherichia coli* and *Pseudomonas* species in a setting of impaired ability of the body to clear the infection. Congenital urinary tract malformations are highly linked with development of XGP in children [13]. Ultrasound and other imaging modalities can be used to suspect XGP, but CT-scan and MRI are thought to be more sensitive and usually show an enlarged nonfunctional kidney with or without invasion to neighbouring structures [14].

On abdominal CT scan, our patient showed foci of calcifications with no calculi but renal calculi (frequently of staghorn proportions) are reported in up to 80 % of cases with XGP.

XGP can manifest with malignant features capable of local tissue invasion and destruction explaining the frequent confusion with renal neoplasm hence it is often referred to as a pseudo tumor [15].

Our case presented with one year history of antibiotic refractory urinary tract symptoms and fever, which is a typical presentation of this condition [16]. The obstruction associated with XGP in the pediatric population is frequently linked to congenital urinary tract malformations than from obstructive calculi but our patient did not have any anomaly or calculi from the imaging performed. Histologically; XGL has a pathognomonic appearance characterized by lipid laden foamy macrophages in a mixture of acute and chronic inflammatory cells [17]. Other laboratory investigations including complete blood count and differential are usually nonspecific but may show leukocytosis and anemia with elevated erythrocyte sedimentation rate.

If XGP is unilateral, serum creatinine and urea are usually normal, except for cases of bilateral extensive disease destroying both kidneys in which case long-term renal replacement therapy is required. Urine analysis reveals leukocytosis, proteins and culture may be positive for bacteria which also helps to ascertain the antibiotic sensitivity pattern [18].

Surgery remains the mainstay for a definitive diagnosis and cure of Xanthogranulomatous pyelonephritis (XGP) requiring extirpation (nephrectomy) as the standard surgical technique but for small tumor, partial nephrectomy can be attempted. In most cases extirpation is necessary because the disease results in an infected, nonfunctioning kidney. Nephron-sparing surgery is an option, especially in cases of bilateral disease with demonstrated significant residual functional renal tissues [19]. Laparoscopic nephrectomy is feasible for some cases of XGP and few cases have been cured by this operative modality and its advantage over radical nephrectomy is being explored. Some pediatric case series of laparascopic resection of XGP have reported promising success with this apporach [20]. The technical difficulty and complications of the procedure raise concern on its wide spread use especially in limited resource settings [21].

Medical therapy with antibitics only has been effective for treatment of XGP in a handful of cases but may be appropriate as a temporary measure for patients requiring workup before nephrectomy and prophylactic antibiotics should be administered before and after surgical intervention. The choice of antibiotic has to be tailored toward the identity and sensitivity of the offending organism. *Proteus* species and *E coli* are usually sensitive to several antibiotics, including first-generation cephalosporins which can be started empirically pending sensitivity results [22]. *Pseudomonas* species has a narrow spectrum of sensitivity and usually require the use of aminoglycosides, third-generation cephalosporins, or fluoroquinolones. For non-septic patients and those evaluated on an outpatient basis, some authors recommend use of oral antibiotics until surgery, at which point intravenous antibiotics should be administered [23]. There is no clear recommendation regarding the duration of antibiotic therapy but data from published case series favor a continuation of oral antibiotics at least for one week post nephrectomy [24, 25]. Our patient underwent left radical nephrectomy and was kept on intravenous Piperacillin-tazobactam for 7 days post nephrectomy and remains asymptomatic at the time of submission of this manuscript.

## Conclusion

This case report highlights the importance of clinicians attending children presenting with fever and renal mass to broaden the differential diagnosis for Wims’ tumor the commonest childhood renal neoplasm to exclude benign tumors, which can be cured by surgical resection and avoid exposure to chemotherapy especially if treatment is to be given using the European (SIOP) guidelines.

## Abbreviations

XGP, Xanthogranulomatous pyelonephritis; CT, computed tomography; WBC, white cell count; RBC, red blood cell; MCV, mean corpuscular volume; MCH, mean corpuscular hemoglobin
